# Genetically Modified Mouse Mesenchymal Stem Cells Expressing Non-Structural Proteins of Hepatitis C Virus Induce Effective Immune Response

**DOI:** 10.3390/vaccines8010062

**Published:** 2020-02-02

**Authors:** Olga V. Masalova, Ekaterina I. Lesnova, Regina R. Klimova, Ekaterina D. Momotyuk, Vyacheslav V. Kozlov, Alla M. Ivanova, Olga V. Payushina, Nina N. Butorina, Natalia F. Zakirova, Alexander N. Narovlyansky, Alexander V. Pronin, Alexander V. Ivanov, Alla A. Kushch

**Affiliations:** 1 Gamaleya National Research Center of Epidemiology and Microbiology, Ministry of Health of the Russian Federation, Moscow 123098, Russia; wolf252006@yandex.ru (E.I.L.); regi.k@mail.ru (R.R.K.); edm95r@rambler.ru (E.D.M.); hyperslava@yandex.ru (V.V.K.); 5893211@bk.ru (A.M.I.); narovl@yandex.ru (A.N.N.); proninalexander@yandex.ru (A.V.P.); vitallku@mail.ru (A.A.K.); 2 Federal State Autonomous Educational Institution of Higher Education I.M. Sechenov First Moscow State Medical University of the Ministry of Health of the Russian Federation (Sechenov University), Moscow 119991, Russia; payushina@mail.ru; 3 Koltzov Institute of Developmental Biology of Russian Academy of Sciences, Moscow 119334, Russia; nnbut@mail.ru; 4 Center for Precision Genome Editing and Genetic Technologies for Biomedicine, Engelhardt Institute of Molecular Biology, Russian Academy of Sciences, Moscow 119991, Russia; nat_zakirova@mail.ru

**Keywords:** hepatitis C virus (HCV), mesenchymal stem cells (MSC), modified MSC, DNA immunization, nonstructural HCV proteins, immune response, HCV vaccine, myeloid derived suppressor cells (MDSCs)

## Abstract

Hepatitis C virus (HCV) is one of the major causes of chronic liver disease and leads to cirrhosis and hepatocarcinoma. Despite extensive research, there is still no vaccine against HCV. In order to induce an immune response in DBA/2J mice against HCV, we obtained modified mouse mesenchymal stem cells (mMSCs) simultaneously expressing five nonstructural HCV proteins (NS3-NS5B). The innate immune response to mMSCs was higher than to DNA immunization, with plasmid encoding the same proteins, and to naïve unmodified MSCs. mMSCs triggered strong phagocytic activity, enhanced lymphocyte proliferation, and production of type I and II interferons. The adaptive immune response to mMSCs was also more pronounced than in the case of DNA immunization, as exemplified by a fourfold stronger stimulation of lymphocyte proliferation in response to HCV, a 2.6-fold higher rate of biosynthesis, and a 30-fold higher rate of secretion of IFN-γ, as well as by a 40-fold stronger production of IgG2a antibodies to viral proteins. The immunostimulatory effect of mMSCs was associated with pronounced IL-6 secretion and reduction in the population of myeloid derived suppressor cells (MDSCs). Thus, this is the first example that suggests the feasibility of using mMSCs for the development of an effective anti-HCV vaccine.

## 1. Introduction

Mesenchymal stem cells (MSCs) are successfully used in various fields of regenerative medicine [1]. Cell therapy is based on the ability of MSCs to migrate to the sites of pathology. They are able to exert anti-inflammatory and immunomodulatory effects upon allogeneic transplantation, as well as in autoimmune diseases [2,3,4,5]. Obtaining genetically modified MSCs expressing introduced genes significantly expands the possibilities of both cellular and genetic therapy, ensuring the delivery of therapeutic molecules to the sites of damage and inflammation [6,7]. MSCs transduced by the interferon β (IFN-β) gene have been shown to reduce the signs of inflammation and the severity of the disease and to improve the condition of the CNS in experimental multiple sclerosis [8]. Positive results with modified MSCs have been obtained in myocardial infarction [9] and in cancer therapy [10].

These results suggest that obtaining and using modified MSCs (mMSCs) that harbor viral genes could be effective for the development of antiviral vaccines. This approach has a number of advantages over traditional vaccine technologies. mMSCs can express many proteins simultaneously, thus ensuring a wide range of epitopes with the correct post-translational modifications as during natural infection. They are also capable of delivering, expressing, and presenting an antigen for a long time. Indeed, Tomchuck et al. demonstrated in an experimental model of HIV infection that cellular vaccines based on transfected MSCs could be developed [11].

One of the urgent but unresolved problems of biology and medicine is the development of an effective vaccine towards hepatitis C virus (HCV). HCV is considered as one of the main etiological agents of chronic liver disease, including terminal stages – cirrhosis and hepatocarcinoma. In up to 80% of cases, acute hepatitis C transfers into chronic disease, which may be caused by a very high heterogeneity of viral genome and the existence of quasispecies, interference of the virus with innate and adaptive immune response pathways, and the formation of “escape” variants of HCV that are not recognized by the immune system [12,13]. Anti-HCV therapy based on a combination of pegylated recombinant IFN-α and ribavirin eliminates the virus in no more than 50% of patients [14]. Modern therapy using direct-acting antivirals (DAA) that target HCV NS3, NS5A, and NS5B proteins makes it possible to cure up to 99% of patients, but the extremely high cost of DAAs makes access to the treatment limited, as exemplified by higher rates of detection of new cases compared to number of patients treated with these drugs [15] Another factor that limits access to treatment is unawareness of a majority (ca. 80%) of patients of their status. In addition, there is a lot of evidence showing that HCV can remain in the liver cells and peripheral blood mononuclear cells (PBMC) of patients after the disappearance of viral RNA in serum, thus establishing occult infection [16,17]. Such ongoing viral replication in hepatocytes can lead to continuous liver injury and may underlie the absence of improvement in clinical outcomes after a sustained virological response achieved in a majority of patients [16].

The development of anti-HCV vaccines can contribute to global efforts to eradicate the virus. Numerous attempts to develop a vaccine whose action is based on broadly neutralizing antibodies against structural proteins failed, most likely due to very high variability of E1 and E2 glycoproteins and escape of virions due to bound lipoproteins and glycans [18]. Efficient vaccines could be based on the recombinant viral proteins/peptides that contain B- and T-cell epitopes or DNA plasmids/viral vectors, ensuring their expression [19]. However, the optimal set of HCV genes or their fragments that should be present in the vaccine has not yet been determined. Literature data suggest that none of the candidate vaccines triggered a full preventive and therapeutic response against HCV [20,21]. Recently, several vaccines against HCV based on dendritic cells (DC) have been reported [22,23]. DCs are highly specialized antigen-presenting cells (APC), so DC-based vaccination based on ex vivo stimulated and matured DCs loaded with HCV specific antigens is an attractive approach to elicit sustained anti-viral response to HCV proteins. However, generation of DCs can require substantial time and expense. It was shown that induction of T cell immune responses by DC vaccination is highly dependent on efficient antigen loading of the DCs [24]. The trials showed that such vaccines did not clear HCV infection in chronic hepatitis C patients despite induction of pronounced T-cell response [25,26]. Thus, the development of MSC vaccines based on genetic and cellular technologies could be one of the most promising strategies to prevent the spread of infectious diseases.

The aim of this work was to obtain MSCs expressing HCV genes and to analyze the humoral and cellular immune responses of animals to the introduction of these modified MSCs. As a model we chose genes of nonstructural HCV proteins that form viral replicase complex, as they are more conservative than structural proteins and in total comprise two-thirds of the entire HCV proteome [27]. Several lines of evidence show that clearance of acute HCV infection in chimpanzees and humans is temporally associated with early, strong, and broadly reactive T cell responses against multiple non-structural viral proteins [12,28]. The non-structural proteins are considered as the dominant targets for CD8+ and CD4+ cells [27]. A robust HCV-specific CD8+ and CD4+ T cell responses to the non-structural proteins has the potential to restrict infection, eliminate virus-infected cells after challenge, and prevent persistent infection at the very least [29].

## 2. Materials and Methods

### 2.1. Animals

Mice of the DBA/2J (H-2d) line (females, 6–8 weeks old) were obtained from the laboratory of the animal breeder Stolbovaya, FMBA, Moscow Region. All animal experiments were carried out in accordance with order 708 of the Ministry of Health of the Russian Federation and with the “Regulations on the ethical attitudes to laboratory animals of N.F. Gamaleya NRCEM (Moscow, Russia)”.

### 2.2. Isolation of Primary MSCs

Mouse primary MSCs were obtained from bone aspirates of DBA mice. Both femurs and tibias from each leg were used. The cell suspension was homogenized and centrifuged at 2000 g for 10 min. Cell pellets were resuspended in high glucose Dulbecco’s Modified Eagle Medium (DMEM) containing 10% fetal calf serum (FCS) (Invitrogen, Waltham, MA, USA), 10 μg/mL insulin, 5.5 μg/mL transferrin, 6.7 ng/mL sodium selenite, 10 ng/mL basic fibroblast growth factor, 2 mM L-glutamine, and 50 μg/mL gentamicin. The cells were seeded in culture flasks (Costar, New York, NY, USA) at a concentration of 2 × 10^6^ cells/mL. The next day, as well as every subsequent 3-4 days, the culture medium was replaced. The resulting adhesive cell population was reseeded using a 0.25% trypsin solution. MSCs were cultured at 37°C in a 5% CO_2_ atmosphere. Unless otherwise specified, culture media and other reagents were purchased from PanEco, Russia (Moscow, Russia).

### 2.3. Characterization of MSCs

Cell morphology and the state of the cell monolayer were examined visually using an AX10 inverted microscope (Zeiss, Germany). Immunophenotypic analysis of MSCs was performed by flow cytometry as described below.

### 2.4. Assessment of Adipogenic and Osteogenic Potencies of MSCs

MSCs isolated from red bone marrow were grown on passage 1 in 12-well plates into an osteogenic medium (growth medium supplemented with 10 nM dexamethasone, 50 μg/mL 2-phospho-L-ascorbate, and 10 mM sodium β-glycerophosphate) at a density of 1 × 104 cells/mL or into an adipogenic medium (standard growth medium supplemented with 10 µM dexamethasone, 0.2 mM indomethacin, 1 IU/mL insulin, and 0.5 mM 3-isobutyl-1-methylxanthine) at a density of 3 × 10^4^ cells/mL. As a control, the cells maintained at the same density in a standard growth medium were used. The medium was changed twice a week; cultivation was continued for 20 days (adipogenesis) or 21 days (osteogenesis). For the analysis of adipogenic differentiation, cells were fixed with 4% formalin in 0.1 M phosphate-buffered saline, pH 7.2-7.4 (PBS), and stained with Oil Red O (Sigma, St. Louis, MO, USA) in order to detect neutral fat inclusions. For the analysis of osteogenic differentiation, the culture was fixed with a mixture of sodium citrate, acetone, and formaldehyde; cytochemical detection of alkaline phosphatase activity was carried out by the method of azo coupling of Fast Red Violet (FRV) with naphthol AS-BI (Sigma, USA), according to the manufacturer’s protocol. In addition, in the culture of MSCs subjected to osteogenesis induction, deposits of calcium salts were detected by staining cells fixed with 96% ethanol with Alizarin Red S (Sigma, USA) at pH 4.1. After cytochemical reactions, the cells of all studied cultures were additionally stained with hematoxylin and analyzed using a Primovert inverted light microscope (Zeiss, Germany).

### 2.5. Plasmid and Transfection of MSC Culture

We used the pcNS3-NS5B plasmid construct encoding five nonstructural HCV proteins (NS3, NS4A, NS4B, NS5A, and NS5B) of genotype 1b that was constructed using a commercially available pcDNA-3.1(+) vector (Invitrogen, USA) [30]. The plasmid was purified from *E. coli* strain *JM109* using a commercial QIAGEN Plasmid Purification Maxi Kit (QIAGEN, Hinden, Germany) according to the manufacturer’s instructions.

To obtain genetically transformed cells expressing HCV proteins, we used a primary MSC culture at third-fourth passages. MSCs were seeded on a six-well plate at a density of 5x10^4^ cells/mL. Twenty-four hours after reaching the subconfluent monolayer (70–90% cells/well), complexes of a plasmid with Xfect Transfection Reagent (Clontech Laboratories, Takara, USA) were applied to the cells. The transformed cells were selected in a medium containing 0.5 mg/mL G-418 (Invitrogen, Waltham, MA, USA). Cell viability was analyzed using a standard MTT test [31] and the trypan blue dye exclusion assay [32]. We conducted several rounds of selection, changing the medium with G-418 every 72 h. Cytokine secretion was measured by quantifying their levels in the conditioned medium.

### 2.6. Immunocytochemical and Immunoblot Detection of Viral Proteins

Expression of HCV proteins in the transfected MSCs was determined by the methods of indirect immunofluorescence and immunoperoxidase staining, using monoclonal antibodies (mAbs) against HCV proteins [33] as primary antibodies and secondary antibodies against mouse immunoglobulins (Ig) conjugated with fluorescein isothiocyanate (FITC) or horseradish peroxidase (HRP) (Dako, Denmark), as previously described [34,35]. Cell nuclei were stained with 4′-6-diamidino-2-phenylindole (DAPI) (immunofluorescence analysis) or with hematoxylin (immunoperoxidase method). The signals were visualized using an Axio Scope A1 microscope (Zeiss, Germany). The proportion of cells expressing viral proteins relative to the total number of cells was counted in at least eight fields of view at a magnification of 400× and expressed as a percentage value. This corresponds to counting at least 1600 cells for each HCV protein.

Western blot analysis was performed as described previously using the same monoclonal antibodies or serum of the rabbits immunized with the respective protein [36].

### 2.7. Immunization of Animals

To study the parameters of the immune response, we used four groups of DBA mice with 10 animals in each group. The mice from group 1 were injected with genetically modified MSCs (mMSC), mice from group 2 with non-transfected, “native” MSCs, mice from group 3 with pcNS3-NS5B plasmid, and mice from group 4 with saline. MSCs and mMSC (5 × 10^5^ cells) were injected into the tail vein, plasmids (100 μg)—intramuscularly into the quadriceps femoris muscle. Two immunizations with an interval of 2–3 weeks were conducted.

In some experiments, the animals were injected with mMSC treated with a recombinant mouse IFN-γ protein (Abcam, Cambridge, UK) at a concentration of 80 ng/mL for 18 h. The immunization scheme was as described above.

### 2.8. The Recombinant HCV Proteins

The recombinant HCV proteins were used as antigens to stimulate T-cell responses in vitro and as sorbents in an enzyme-linked immunosorbent assay (ELISA) to evaluate antibody production. The proteins were combined into four pools: NS3 (protease domain with a sequence of 1027–1229 aa, helicase domain 1230–1658 aa, immunodominant region 1356–1459 aa, genotype 1b); NS4 (1677–1754 aa and mosaic protein containing regions 1691–1710, 1712–1733, 1921–1940 aa from genotypes 1, 2, 3, and 5); NS5A (the full-length protein 1973–2419 aa and fragments 2061–2302 aa, 2212–2313 aa, genotypes 1b and 1a); the NS5B protein lacking C-terminal hydrophobic 21 amino acid residues (2420–2990 aa, genotype 1b); as a negative control, we used the nucleocapsid (core) protein (1–90 aa). The recombinant proteins were expressed in *E.coli* and purified by chromatography on Ni-NTA-agarose or on glutathione sepharose, as described previously [30,37,38,39].

### 2.9. Humoral Immune Response

The immune response to the injected constructs was assessed 10 days after the second immunization. The activity of antibodies against HCV proteins in mouse sera was determined by indirect ELISA, as described previously [30]. As secondary antibodies, we used antibodies against mouse Ig isotypes IgG1 and IgG2a conjugated to HRP (Jackson Immunoresearch Laboratories, Cambridge, UK). As the serum titer in ELISA, we used the reciprocal of the highest serum dilution, at which the optical density was 2 times higher than that for the control group.

### 2.10. T-Cell Proliferation and ELISpot Assays

T-cell proliferation was assessed by the incorporation of [^3^H]-thymidine into the DNA of dividing cells. The spleens of 10 mice of each group were pooled, a suspension of splenocytes was seeded in U-bottomed 96-well microculture plates at a density of 5 × 10^5^ cells/well, and specific stimulants (pools of the recombinant HCV NS3, NS4, NS5A, and NS5B proteins at a final concentration of 1 μg/mL) were added. As negative controls, we used medium alone (spontaneous proliferation) and recombinant HCV core protein. Mitogen concanavalin A (ConA, 5 μg/mL, Sigma, USA) was used as an unspecific positive control. All samples were set in at least three replicates. The cells were cultured in a RPMI-1640 medium containing 20% FCS (Invitrogen, USA), 4.5 mg/mL glucose, 2 mM glutamine, 0.2 u/mL insulin, and 50 μg/mL gentamicin at 37 °C in a 5% CO_2_ atmosphere. Four days later, aliquots of cell culture fluids were withdrawn and frozen at −20 °C. The remaining cells were labeled with 1 μCi/well [^3^H]-thymidine (TdR, Amersham-Pharmacia-Biotech) and 18 h later harvested onto the glass-fiber filters. The radioactivity was measured using a MicroBeta2 β-counter (PerkinElmer, Waltham, MA, USA). Results were expressed as stimulation indexes (SI), determined by dividing the mean radioactive ^3^H incorporation as counts per minute (c.p.m.) in the presence of antigens by means of ^3^H incorporation in the wells containing medium alone and control antigen.

The number of IFN-γ synthesizing cells was determined using the ELISPOT mouse IFN-γ Kit (BD Biosciences, San Jose, CA, USA) according to the manufacturer’s instructions. The results were expressed as the number of spot forming cells (SFC) per 10^6^ cells.

### 2.11. Detection of Cytokines in Cell Culture Fluids by Sandwich ELISA

Measurement of cytokine levels (IFN-γ, TNF-α, IL-2, IL-6, IL-10, IL-12) was performed by ELISA in conditioned medium from selection of the MSC cells transfected with pcNS3-NS5B. The concentration of IFN-γ was also determined in medium from the splenocytes stimulated for four days. We used the Mouse IL-6 ELISA development kit (HRP), Mouse IFN-γ ELISA development kit (HRP), Mouse TNF-α ELISA development kit (HRP) (Mabtech, Stockholm, Sweden), Mouse IL-10 DuoSet ELISA, and Mouse IL-12 p70 Duoset ELISA (R&D Systems, Minneapolis, MN, USA). The detection sensitivity for IL-6 was 10 pg/mL, for IFN-γ and TNF-α—2 pg/mL, for IL-2—4 pg/mL, for IL-10 and IL-12—30 pg/mL. The concentrations of cytokines were determined from the calibration curves of standard samples.

### 2.12. Flow Cytometry

For immunophenotyping of MSCs, adhesive cells of the 2nd-3rd passages were collected using Accutase (CLS, Eppelheim, Germany), washed twice in ice-cold PBS, and separately stained (10^6^ cells/sample) with primary phycoerythrin (PE)-labeled antibodies against CD73, CD90.1, and CD105 (BD Biosciences, USA) for 45 min, or unlabeled rat antibodies against CD45 and CD34 for 45 min and secondary Rabbit Anti-Rat-FITC conjugate (Abcam, Cambridge, UK) for 30 min at room temperature.

Analysis of dendritic (DC) and myeloid derived suppressor cells (MDSCs) was performed by multicolor flow cytometry. The suspensions of splenocytes from 10 immunized mice of each group (10^6^ cells/sample) were incubated with PE-labeled antibodies against CD11c, FITC-labeled antibodies against Gr-1 (Ly-6G and Ly-6C), and allophycocyanin (APC)-labeled antibodies against CD11b (BD Biosciences, USA). Antibodies to the corresponding isotype controls were included in all experiments. The absolute and relative number of cells carrying the markers was assessed by FACS on a flow cytometer BD FACSCanto II (Beckton Dickinson, Franklin Lakes, NJ, USA) using free software BD FACSDiva, v.6.1.3 (BD Biosciences, San Jose, CA, USA).

### 2.13. Determination of Type I IFNs (IFN-α/β) Production by Immune Cells

Quantification of IFN-α/β was carried out by a biological method in accordance with techniques based on the antiviral effect of interferons [40,41]. Briefly, mice splenocytes and peripheral blood leukocytes from 10 immunized mice of each group were stimulated in vitro by Newcastle disease virus (a standard IFN-α/β inducer) [42,43]. After virus inactivation, serial dilutions of the culture fluids were added to mouse fibroblast L-929 cell culture and 24 h later infected with encephalomyocarditis virus (EMCV). IFN-α/β activity was estimated as the highest reciprocal dilution that caused a 50% decrease in the cytopathogenic effect of 100 tissue culture infectious doses (TCID5O) per ml, and was expressed in international units per ml (IU/mL) using WHO International Standard Interferon alpha-2b (NIBSC cat. #95/566: https://nibsc.org/products/brm_product_catalogue/who_standards.aspx).

### 2.14. Phagocytic Activity

Phagocytic activity was determined by quantification of reactive oxygen species (ROS) production by the luminol-dependent chemiluminescence (CL) method. As a CL activator, we used opsonized zymosan, as described previously [44]. For analysis, we used a suspension of mice splenocytes and peripheral blood leukocytes from 10 immunized mice of each group. Spontaneous and induced CL were measured on a Synergy H1 hybrid multifunction photometer (BioTek, USA). As a quantitative indicator of the level of ROS production, we used an activation index (AI) – the ratio of the intensity of induced CL to the intensity of spontaneous CL.

### 2.15. Statistical Analysis

Statistical analysis was performed with Statistica 6 software (StatSoft Inc., Tulsa, OK, USA). Prism 5 software (GraphPad5, SanDiego, USA) was used to create graphs. Data was presented as means ± SD (standard deviation) of three independent experiments and analyzed by two-tailed Student t-test or one-way analysis of variance (ANOVA), followed by Tukey tests for multiple comparisons, when appropriate (*p* < 0.05 was considered as statistically significant).

## 3. Results

### 3.1. Cells Isolated From the Bone Marrow of Mice Display Features of Mesenchymal Stem Cells

**Figure 1:**
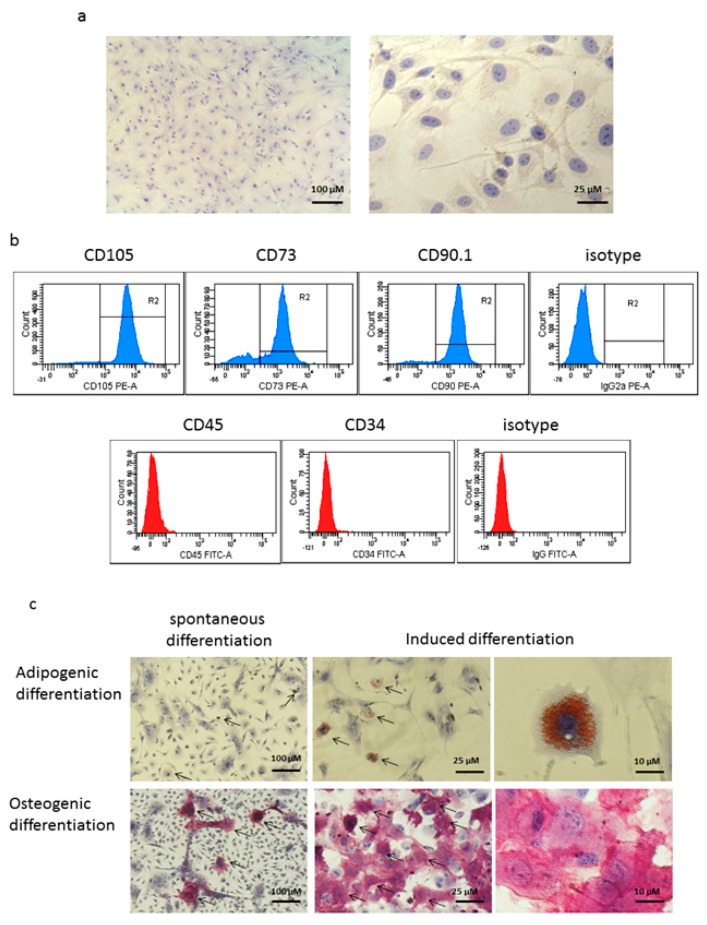
Characterization of mesenchymal stem cells (MSC) isolated from the bone marrow of mice. (**a**) The morphology of MSC isolated from the red bone marrow of mice is polymorphic cells that exhibit a fibroblast-like shape. The cells were seeded on culture flasks, and, after staining the nuclei with hematoxylin, they were visualized by light microscopy, scale bar, 100 µM (left), and 25 µM (right). (**b**) These are typical results of MSCs receptor analysis by flow cytometry. For immunophenotyping of MSCs, adhesive cells of the second and third passages were stained with phycoerythrin (PE)-labeled antibodies against CD73, CD90.1, CD105, or PE-labeled antibodies of the corresponding isotype controls, and unlabeled rat antibodies against CD45 or CD34 followed by rabbit antirat fluoresceine isothiocyanate (FITC) conjugate. The isolated MSCs expressed CD105, CD73, and CD90 (upper panel), but did not express CD45 and CD34 (lower panel). (**c**) The adipogenic (upper panel) and osteogenic (lower panel) differentiation of MSC isolated from the bone marrow of mice. MSCs were grown into an adipogenic or osteogenic medium; the medium was changed twice a week for 20 days (adipogenesis) or 21 days (osteogenesis). Then, MSCs were stained to detect neutral fat inclusions or alkaline phosphatase activity, respectively. Single adipocytes (upper left panel) and osteocytes (lower left panel) were present in the control cultures; arrows indicate spontaneous differentiation, scale bar, 100 µM; induced cultures MSC, arrows indicate fat cells (middle and upper right panel) and osteocytes (middle and lower right panel), (scale bar, 25 µM, and 10 µM, respectively).

MSCs isolated from the bone marrow of mice were characterized by adhesiveness and morphology. Light microscopy showed that MSCs were attached to the surface of culture flasks and were polymorphic cells with a fibroblast-like morphology. Cells actively proliferated and formed a monolayer (Figure 1a).

Phenotyping of MSCs of two to three passages by flow cytometry showed that most of them (83–96%) expressed CD73, CD90.1, and CD105 receptors that are markers of MSCs (Figure 1b, upper panel). At the same time, no expression was detected for hematopoietic cell markers CD45 and CD34 (Figure 1b, lower panel).

Analysis of the adipogenic potency of the obtained MSCs revealed that, at the end of 20 days of cultivation, single adipocytes were present in the control culture (Figure 1c, upper left panel); adipocytes located either individually or in small, usually loose groups of 5–10 cells were detected in the induced culture (Figure 1c, middle and upper right panel). Adipocytes differed in morphology and were at different stages of fat droplet accumulation: both mature oval or round adipocytes containing many large lipid vacuoles were found, as well as fibroblast-like cells, in which fat inclusions were smaller and occupied only part of the cytoplasm.

A study of the osteogenic differentiation of MSCs showed that, after 21 days of cultivation, the cells in the control culture formed a confluent monolayer of uneven density with cells of fibroblast-like morphology. The cytochemical reaction to alkaline phosphatase, a marker of osteogenic cells, identified just a few cells containing this enzyme in the control culture (Figure 1c, lower left panel). In contrast, in the induced culture, most cells that contained alkaline phosphatase significantly exceeded those in the control culture (85±13 vs. 9±8, respectively, p<0.001). Cells with the positive reaction had a fibroblast-like shape and formed clusters of various size and density, in many cases closely contacting each other. The reaction intensity varied in different cells and often was very high (Figure 1c, middle and lower right panel), thus showing that the cells underwent osteogenic differentiation. Therefore, MSCs isolated from mouse bone marrow exhibited moderate capability to differentiate into mature multilocular adipocytes. They also could pass initial stages of osteogenic differentiation. It can be concluded that the isolated and multiplied cells corresponded to the generally accepted minimal characteristics of MSCs [45].

### 3.2. Modified MSCs Express Genes of Hepatitis C Virus

**Figure 2:**
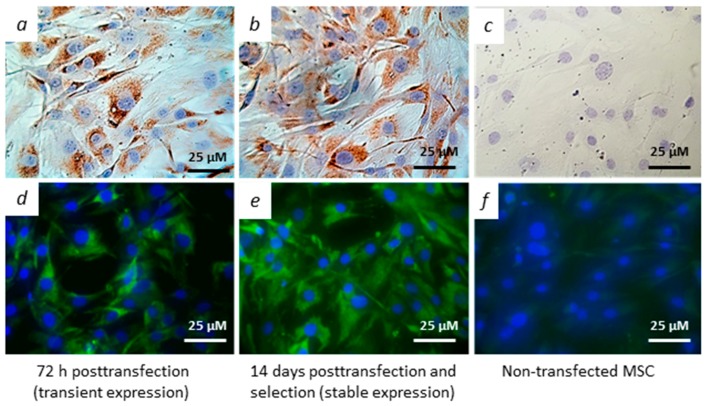
Immunocytochemical staining of hepatitis C virus (HCV) NS5A in MSCs transfected with pcNS3-NS5B. Primary MSC cultures at third-fourth passages were transfected with the pcNS3-NS5B plasmid and later selected with G-418. The cells were stained with monoclonal antibody against HCV NS5A using immunoperoxidase (top panel) and immunofluorescence (bottom panel) methods. NS5A is located in cytoplasm (brown or green, respectively); nuclei were stained with hematoxylin or 4′,6-diamidino-2-phenylindole (DAPI), respectively (blue). (**a**,**d**) 72 h post-transfection; (**b**,**e**) two weeks post-transfection and selection; (**c**,**f**) non-transfected MSC (scale bar, 25 µM).

MSCs were transfected with the pcNS3-NS5B plasmid and the expression of viral genes was analyzed by immunocytochemical methods. Transfection efficiency was 55% on average. Figure 2a,d show typical staining of the transfected cells with monoclonal antibody against NS5A protein; expression of the other target HCV proteins was also demonstrated (Figure S1). The cell lines, obtained by selection of the transfected cells in the presence of G-418 for two weeks, also demonstrated expression of the HCV proteins (Figure 2b,e), in contrast to the non-transfected MSC (Figure 2c,f). Finally, expression of the HCV proteins was also confirmed by western blotting (Figure S2).

### 3.3. Dynamics of Cytokine Production in Transfected MSCs

**Figure 3:**
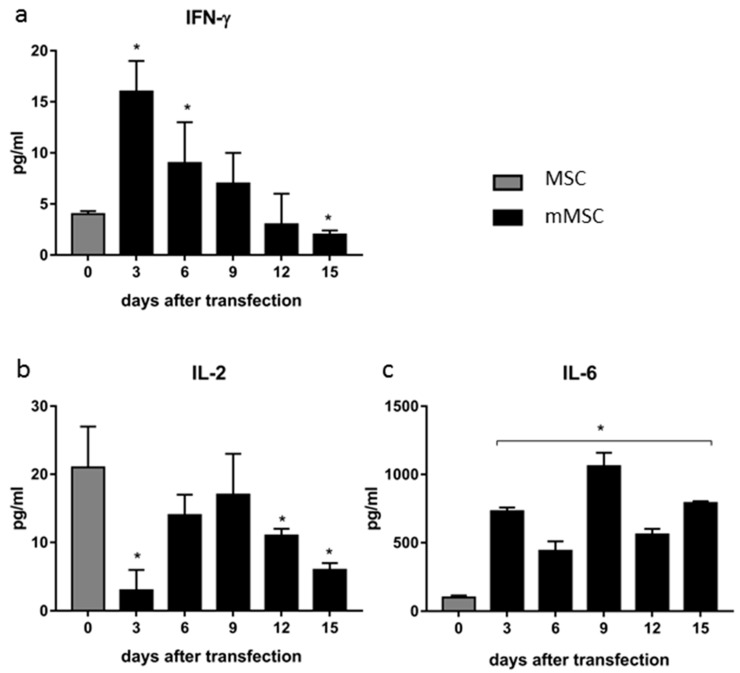
Levels of cytokines secreted by MSC in vitro after transfection with pcNS3-NS5B. The MSCs were transfected with pcNS3-NS5B, selected with G-418 for two weeks with changing medium every three days, and cytokine levels were quantified in a conditioned medium. The concentrations of IFN-γ (**a**), IL-2 (**b**), and IL-6 (**c**) are expressed in pg/ml. Values on each diagram are means ± SD of four measurements done in three independent experiments, * *p* < 0.05 compared to the non-transfected MSC (gray bars).

Next, we measured levels of cytokines IL-2, IL-6, IL-10, IL-12, IFN-γ, and TNF-α secreted by MSCs in vitro after transfection of cells with pcNS3-NS5B and sequential selection using G-418. The nontransfected MSCs secreted three cytokines: IFN-γ, IL-2, and IL-6, whereas the other cytokines were not detected in the conditioned medium. The concentration of IFN-γ increased up to the third day post-transfection, and then gradually decreased until the limit of detection at day 15 (end of cell selection) (Figure 3a). Different kinetics was observed in the case of IL-2: after an initial decrease in its level at day 3, its levels were increased at days 6–9 with a second decrease afterwards (Figure 3b). On the contrary, secretion of IL-6 was significantly increased during the entire observation period and exceeded by eightfold at day 15^th^ the levels in the case of the untransfected MSCs (Figure 3c).

### 3.4. Immune Response to Administration of Modified MSCs to Mice Exceeds Immune Response to Plasmid

Our next goal was to evaluate the immune response in mice immunized with the mMSC. Comparative analysis of the humoral immune response in mice showed that mMSC (group 1) induced the formation of antibodies to all viral proteins encoded by the plasmid. Levels of antibodies to NS3, NS4, and NS5A were on average 40-fold higher than in group 3 (immunized with the pNS3-NS5B plasmid) (Table 1). In contrast, levels of antibodies to NS5B were higher in mice immunized with the plasmid than with mMSC (Table 1). The distribution of antibody isotypes for HCV proteins differed between the groups: in groups 1 and 3, the antibodies belonged mainly to the IgG2a isotype; in group 1, IgG1 antibodies to the NS3 protein were also detected. After introduction of naïve MSCs (group 2), IgG2a antibodies were not found, while IgG1 antibodies were detected in only four out of ten mice and were present at a low level. Therefore, differences with the control were not statistically significant.

To assess the cellular response of lymphocytes in vitro, we used recombinant proteins from the HCV non-structural region as specific stimulants. For a negative control, we also assessed the response to the core protein that was not encoded by the pNS3-NS5B plasmid and in mMSCs. For a positive control, ConA was used for stimulation.

**Figure 4:**
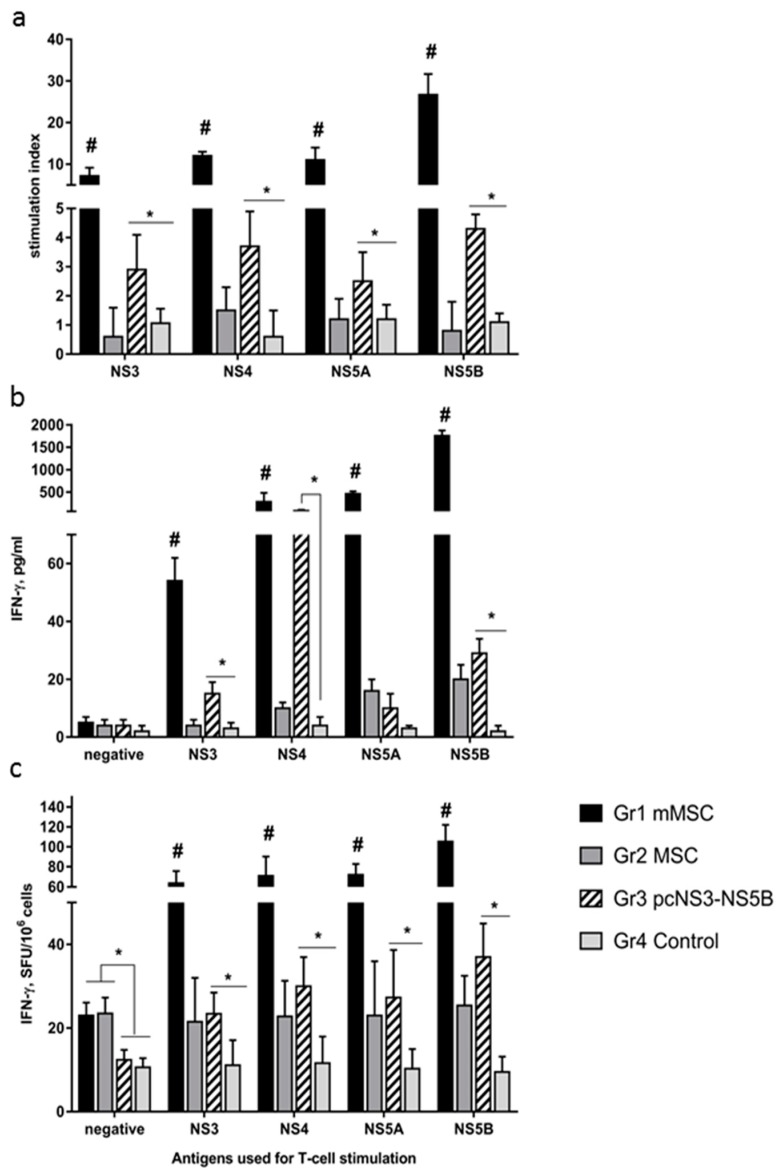
A comparative analysis of the cellular immune response in mice to HCV proteins in vitro after immunization with modified MSC, naïve MSC, and plasmid. Four groups (Gr) of mice were injected twice with mMSC (Gr1), non-transfected MSC (Gr2), plasmid pcNS3-NS5B (Gr3), or saline (Gr4). To assess the cellular response of lymphocytes in vitro, we used purified recombinant proteins from the non-structural region of HCV, which were combined into four pools (NS3, NS4, NS5A, and NS5B). A recombinant HCV core and medium alone were used as negative controls (negative). (**a**) Results of T-cell proliferation are expressed as stimulation indexes (SI); the IFN-γ production by splenocytes in response to HCV antigens was assayed as cytokine concentrations in culture fluids by ELISA, expressed as pg/ml (**b**), or as the number of IFN-γ synthesizing cells by ELISpot in the number of spot forming cells (SFC) per 10^6^ cells (**c**). Values on each diagram are means ± SD of three measurements done in three independent experiments. * *p* < 0.05 compared to control; # *p* < 0.05 compared to all groups.

All tested non-structural HCV proteins stimulated proliferation of splenocytes in groups 1 and 3, SIs statistically significantly (*p* < 0.05) differed from SIs in groups 2 and 4 (Figure 4a). In group 1, SIs exceeded those in group 3 by 2.5–6.1 times, on average by 4.2 ± 1.6 times. The greatest proliferative response was caused by NS5B, when SI reached 27.

Stimulated lymphocytes secreted IFN-γ (Figure 4b). In group 1, the response was obtained to all specific antigens; the highest cytokine concentration (over 1.7 ng/mL) was stimulated by NS5B. In group 3, the production of IFN-γ was induced by all proteins except NS5A; the NS4 protein showed the highest activity. In groups 1 and 3, the cytokine levels secreted in response to specific antigens varied in a wide range from 3.5 to 60-fold, being on average 30-fold higher than in the case of groups 2 and 4.

In ELISpot assay, the average number of IFN-γ synthesizing cells in response to four specific stimulants in groups 1 and 3 was significantly higher than that in the control groups (Figure 4c). Differences in signal intensity between groups 1 and 3 were 2.6 ± 0.2. The NS5B protein exhibited higher activity than other virus antigens. It should be noted that group 2, which was administered to naïve MSCs, demonstrated immune responses to HCV proteins in contrast to group 4, but their intensity was much lower than in the case of the transfected cells in group 1 (Figure 4b,c).

The ELISpot reaction was also performed with splenocytes from mice that were administered mMSC treated with IFN-γ. A number of IFN-γ producing cells in this group was 11-fold lower than those in group 1. It is noteworthy that the number of spots for NS3 was 13.3 ± 7.1%, for NS4—7.1 ± 2.6%, for NS5A—7.6 ± 1.5%, for NS5B—8.9 ± 4.2% of the corresponding values in group 1.

Assessment of the results of T-cell proliferation and ELISpot assays showed that mouse groups 1 and 2, which received MSCs, had a higher level of spontaneous cellular response as well as the response to ConA, compared to groups 3 and 4 (*p* < 0.05, Table 2). Differences in IFN-γ production during ConA stimulation between groups were not statistically significant.

### 3.5. Decrease in Proportion of Myeloid Derived Suppressor Cells in Spleens of Mice Immunized with MSCs

**Figure 5:**
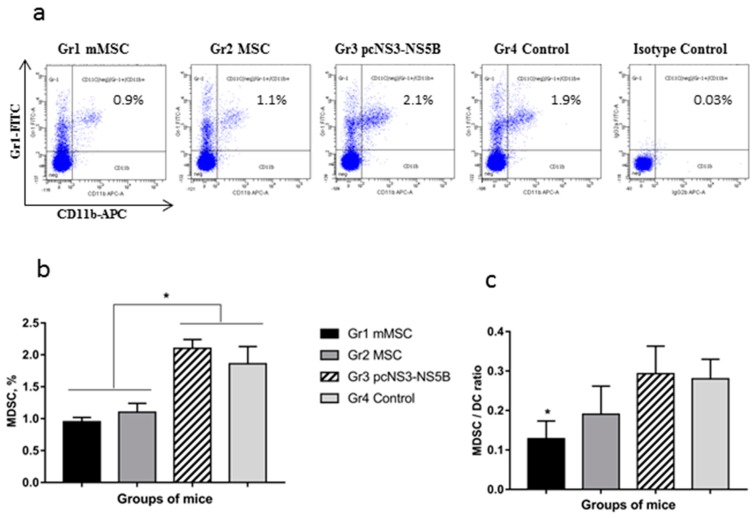
A comparative analysis of the proportion of myeloid derived suppressor cells in spleens of immunized mice. Splenocytes from the immunized mice were stained with anti-CD11c, anti-CD11b, and anti-Gr1 antibodies and analyzed by flow cytometry. Representative dot plots (**a**) and mean values (**b**) of the number of MDSC expressing CD11b and Gr1, and negative for the marker of DC (CD11c) are shown, in the percentages; (**c**) the MDSCs to dendritic cells (DC) ratio. Values on each diagram are means ± SD of three independent analysis, and each of them was performed in triplicate. * *p* < 0.05 compared to Gr3 and Gr4 (**b**) or compared to the control Gr4 (**c**).

Using flow cytometry, we compared the number of splenocytes from immunized mice that express CD11b+Gr-1+ markers of MDSCs and do not express CD11c marker of dendritic cells (DC) (Figure 5a). An almost twofold decrease in the relative content of MDSCs in groups 1 and 2 compared with groups 3 and 4 was found (*p* = 0.007 and 0.015, respectively) (Figure 5b). The content of CD11c+ dendritic cells varied from 6.3 to 7.5% and did not differ between groups (results not shown, *p* > 0.05). The lowest MDSCs to dendritic cells ratio was observed in the spleens of mice of group 1 (*p* = 0.003 compare to control) (Figure 5c).

### 3.6. Changes in Activity of IFN-α/β and in Phagocytic Activity of Immune Cells of Immunized Mice

**Figure 6:**
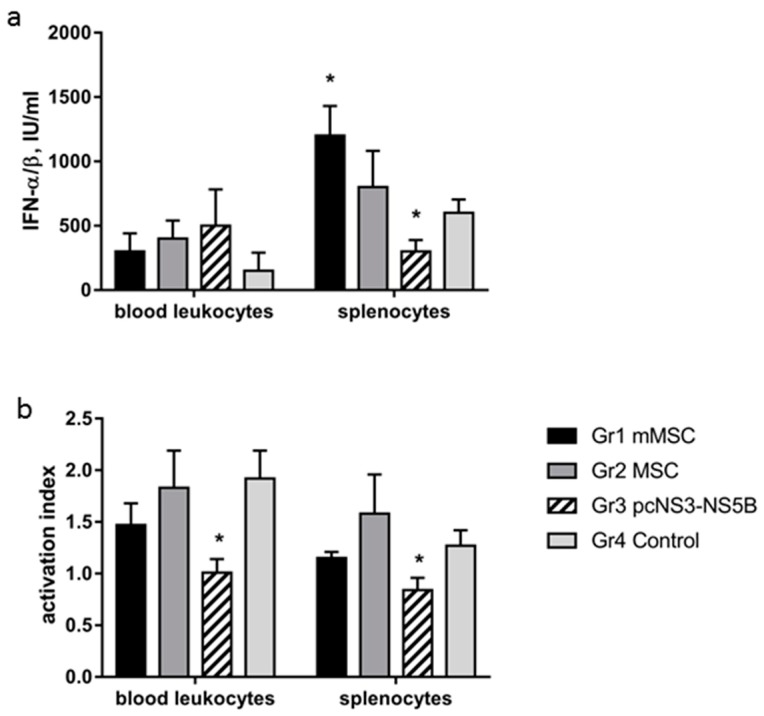
The differences in the biological activity of type I IFNs and phagocytic activity of peripheral blood leukocytes and splenocytes from immunized mice. (**a**) The activity of type I IFNs was evaluated by the production of IFN-α/β in response to stimulation of leukocytes by the Newcastle disease virus in vitro and was expressed in IU/ml; (**b**) phagocytic activity of immune cells was determined by luminol-dependent chemiluminescence (CL) and was expressed as activation index—the ratio of the intensity of induced CL to the intensity of spontaneous CL. The values on each diagram are means ± SD of six measurements done in three independent experiments. * *p* < 0.05 compared to control group 4.

Next, the biological activity of type I IFNs and the phagocytic activity of leukocytes in groups of the immunized mice were studied. Activity of type I IFNs was evaluated by measuring relative production of IFN-α/β in response to stimulation of leukocytes by the Newcastle disease virus. There was no statistically significant difference in the production of IFN-α/β by peripheral blood cells between the groups. Mouse splenocytes more actively produced type I IFNs; the highest production was found in group 1, whereas the smallest was in group 3 (Figure 6a).

Phagocytic activity between the groups was compared by quantification of the production of reactive oxygen species (ROS), determined by luminol-dependent chemiluminescence (CL). For analysis, we used a suspension of splenocytes and a leukocyte preparation from heparinized whole blood. The activation index (AI) was reduced in the blood and in splenocytes of group 3 mice (Figure 6b) due to an increase in the level of spontaneous CL and a decrease in induced CL. Spontaneous CL shows the level of ROS production by cells, and an excessive increase in CL could be associated with a hazardous effect of ROS towards the cells. Induced CL reflects a potential ability of cells to respond to stimuli. The inability of immune cells to stimulate CL (AI ≤ 1) can point to inhibition of the bactericidal activity of phagocytes upon immunization with a plasmid. In the remaining experimental groups, the AI values were >1 and did not differ from the control group.

## 4. Discussion

Advances in cell therapy in recent years are associated with the use of the immunosuppressive properties of MSCs in transplantology, oncology, and some other areas of medicine, although many issues remain unresolved [46]. Depending on the microenvironment, MSCs can exhibit both immunosuppressive and immunostimulating properties [47,48]. However, the mechanisms of stimulation of the immune response by MSCs remain vague and have been studied mostly in vitro in mixed leukocyte reactions (MLR) [47,49].

To date there are only two papers investigating immune response to MSCs that express viral proteins. The first one describes the cells that express human immunodeficiency virus (HIV) Gp120 [11]. The second, recently published by Bolhassani et al. (2019) showed immunization of mice by mMSC that express E7 protein of human papillomavirus (HPV E7 antigen) in a complex with small heat shock proteins leads to a strong T-cell immune response and to a partial protection of animals against HPV-induced tumors [50]. Our data present a first evidence that modified MSCs (mMSCs) expressing HCV proteins affect innate and adaptive immune responses in mice. The experimental data show that the administration of naïve or modified MSCs to mice increases both spontaneous and ConA-induced levels of lymphocyte proliferation. Assessment of the proliferative response to mitogens is one of the most universal tests to assess the lymphocyte function; a weak reaction indicates the failure of cellular immunity. The functional activity of activated lymphocytes (production of IFN-γ) increased. The biological activity of IFN-α/β increased, as established in the test with the inhibition of the cytopathogenic effect of the encephalomyocarditis virus. Type I IFNs are known to play an important immunoregulatory role in relation to both the innate and adaptive immune responses to viral infections. For example, IFN-α/β induces the cytotoxicity of NK cells and increases the expression of MHC class I and co-stimulating molecules on antigen-presenting cells (APCs) [51]. A comparison of the effectiveness of these parameters of the innate immune response to mMSC with the response to immunization with a DNA construct shows that in the latter case the immune cells induce IFN-α/β and IFN-γ at a lower level. When mice were immunized with DNA, the phagocytic activities of monocytes/macrophages, neutrophils and dendritic cells decreased as well. In contrast, the functions of phagocytic cells during immunization with MSCs and mMSCs remained at the level of healthy intact mice.

The adaptive immune response to mMSCs was also significantly higher than that to the plasmid. For instance, mMSC induced a humoral response to all viral proteins expressed in MSCs. Almost all antibodies to non-structural HCV proteins were of IgG2a isotype. Switching to the synthesis of IgG2a antibodies is controlled by the Th1 cellular component of the immune response: a correlation between the level of IgG2a, virus-neutralizing properties, and IFN-γ synthesis has been established [52,53]. The DNA construct also caused the formation of IgG2a antibodies, but their activity was less than that in response to modified MSCs. The most significant difference in antibody titers (40-fold) was found in response to HCV NS3 and NS4 proteins. It should be noted that in combination with the gene adjuvant, pcGM-CSF plasmid, the pcNS3-NS5B construct induced a more active formation of IgG2a antibodies [30]. The immunomodulatory orientation of MSCs with respect to the B-cell response has been suggested to depend on the level of stimulation with viral antigens: the weaker the signal, the greater the stimulating potencies of MSCs [54]. A high level of IL-6 produced by mMSC can stimulate an active humoral response to HCV: this cytokine has been shown to be necessary for differentiation of B cells and secretion of immunoglobulins [54,55].

Production of IgG1 antibodies against HCV was detected in groups immunized with the non-transfected MSCs; however, these antibodies were detected sporadically and with low activity. This may be caused by a cross-reaction between the recombinant proteins obtained in the bacterial system and the antibacterial antibodies in the blood sera of mice.

We characterized the cellular response by the proliferation of lymphocytes and their functional activity—secretion and intracellular content of IFN-γ. These methods are considered as the most informative by a majority of authors [17]. The cellular immune response to the modified MSC significantly exceeded the response to the plasmid pcNS3-NS5B in the proliferative response to the HCV sequence, as well as in the synthesis and secretion of IFN-γ. Differences in the signal intensity between groups 1 and 3 were 2.6–4.2 times in T-cell proliferation and ELISpot and up to 30 times in ELISA when determining the concentrations of produced IFN-γ. All non-structural HCV proteins elicited a cellular response; the maximum response was observed for NS5B. This protein is an RNA-dependent RNA polymerase, the key component of HCV replicase. NS5B is a target for direct-acting antivirals in the treatment of hepatitis C [56]; NS5B has the largest number of conserved T-cell epitopes that are important for vaccine design and induction of an effective immune response [57]. Thus, when immunizing with mMSCs, we achieve a functionally active T-cell response to several HCV proteins simultaneously, including different genotypes. This result is very important as an HCV vaccine should elicit multiantigenic, multigenotypic responses that should protect against challenge with the range of genotypes and subtypes circulating in the community [29]. We also hope that usage of more conserved viral proteins (i.e. nonstructural) will allow induction of a pangenotype response.

One of the mechanisms of the suppressor action of MSCs on the adaptive immune response in inflammation and cancer is believed to be their inhibition of maturation of antigen-presenting dendritic cells under the action of various soluble factors - TGF-β, IL-10, NO, and PD-1 [48]. We showed that when immunizing healthy animals in all experimental groups, the number of dendritic cells did not change compared to the control.

A very interesting fact is the data that immunization with MSCs and mMSC causes a twofold decrease in the number of MDSCs, a heterogeneous population of immature myeloid cells with a powerful suppressor potential. This phenomenon for immunization with MSCs is described for the first time. The role of MDSCs in viral infections has not been adequately studied [58]. In patients infected with HCV, an increase in the MDSC population is observed; these cells inhibit the proliferation of CD4^+^ and CD8^+^ lymphocytes, NK cells, and IFN-γ production [59,60]. Similar results have been obtained in the study of cells from patients with HIV and hepatitis B infections [61,62]. Most experimental data show that the administration of MSCs in oncological and autoimmune diseases results in MDSC accumulation and immunosuppression mediated by certain chemokines and growth factors [63,64,65]. On the other hand, when modeling cancer in mice, a dependence of the immunomodulatory “phenotype” of MSCs on the injection site was found: the simultaneous injection of MSCs with tumor cells led to immunosuppression, distal injection led to immunostimulation; the immune response was shown to correlate with a decrease in the proportion of MDSCs and T-regulatory cells (Treg) [66]. Thus, one of the mechanisms of stimulation of the innate and adaptive immune response to naïve and modified MSCs in our experiments may be the suppression of MDSCs.

During inflammation, the immunosuppressive properties of MSCs are manifested: they suppress both innate and adaptive immunity, weakening the maturation and ability to present antigens by dendritic cells, inducing the polarization of macrophages in the direction of the alternative phenotype, inhibiting the activation and proliferation of T and B lymphocytes and reducing the cytotoxicity of NK cells [48]. We administered MSCs to healthy mice. On the one hand, the biological properties of MSCs depend on the microenvironment; on the other hand, the immunomodulating effect of MSCs themselves is mediated by the secretion of various soluble factors. We compared the production of several cytokines by naïve and modified MSCs in vitro. It turned out that the expression of HCV proteins influenced the production of at least three pro-inflammatory cytokines - IFN-γ, IL-2, and IL-6. For example, after two weeks of cultivation of transfected cultures of MSCs in the presence of G-418, the concentrations of secreted cytokines IFN-γ and IL-2 decreased by 2–3.5 times, and that of IL-6 increased by eight times compared with MSCs. These stably transfected cells were injected into mice. Interestingly, the accumulation of IL-6 leads to the activation of the pro-inflammatory phenotype of the MSC population—MSC-1 [47] and promotes the formation of Th17 cells that activate the immune response [67]. Most likely, fluctuations in cytokine production are associated with the action of viral proteins on cell metabolism but not with changes in MSCs epigenetics because mesenchymal cells have been shown to maintain the genetic stability for at least seven to nine passages [68]. The spectrum of cytokines that are produced by cells transfected with HCV genes has been previously found to change with time, depending on the type of cells and specific viral proteins [69,70,71].

The data on the effect of exogenous IFN-γ on MSCs are contradictory. Several authors have noted an increase in the antigen-presenting properties of MSCs as a result of IFN-γ pretreatment [72]. Other studies have shown that the “priming” of MSCs in vitro with IFN-γ, TNF-α, or IL-1β leads to the formation of the immunosuppressive phenotype MSC-2 [47,73,74]. Our results showed, for the first time, that after administration in mice, modified MSCs treated with IFN-γ cause a pronounced (tenfold) decrease in the cellular response. This means that an excessive concentration of pro-inflammatory cytokines does not stimulate, but instead inhibits the immune response to antigens presented by MSCs.

The major technique that is recommended for evaluation of T-cell response to novel HCV vaccines is quantification of IFN-γ production by ELISpot assay that shows activity of antiviral response [19,75,76]. Though we have not shown protection against HCV infection using mMSCs, such experiments could be performed in future using the respective models. So, we consider our results as a basis for subsequent preclinical (and clinical) studies of protective effect of mMSCs in future. Human mMSCs that express non-structural HCV proteins could be evaluated as a prophylactic vaccine that triggers a strong T-cell response. A growing trend in human MSC clinical trials is the use of allogenic and culture-expanded cells [1]. Application of mMSC to chronic hepatitis C patients may enhance therapeutic response to direct acting antivirals (DAA) via enhanced T-cell immune response that can clear the infected cells. Studies of a combined usage of various candidate HCV vaccines and antiviral agents including DAA are one of the current trends in the field (as can be exemplified by [77,78]. Despite the fact that the results do not always show enhanced clearance of the infection, this approach is still considered promising [19,79,80].

## 5. Conclusions

Thus, for the first time, we demonstrated the feasibility of using modified MSCs expressing non-structural HCV proteins as a platform for creating an effective vaccine against hepatitis C. The mMSC induced a higher innate and adaptive immune response than DNA immunization with the same plasmid. The immunostimulating phenotype of these cells is associated with a high level of IL-6 secretion and a reduction in the proportion of myeloid-derived suppressor cells.

## Figures and Tables

**Table 1:** The levels of anti-HCV antibodies in the sera of mice receiving two injections of mMSC, MSC, or plasmid.

HCV Proteins	Group 1 mMSC		Group 2 MSC		Group 3 pcNS3-NS5B		Group 4 Control	
	IgG1	IgG2a	IgG1	IgG2a	IgG1	IgG2a	IgG1	IgG2a
NS3	200 ± 34 #	410 ± 27 #	20 ± 14	<10	<10	10	<10	<10
	(4/10)	(10/10)	(2/10)	(0/10)	(0/10)	(0/10)	(0/10)	(0/10)
NS4	<10	1600 ± 24 #	80 ± 63	<10	90 ± 64	40 ± 12 *	<10	<10
	(0/10)	(10/10)	(4/10)	(0/10)	(2/10)	(9/10)	(0/10)	(0/10)
NS5A	<10	40 ± 7 #	10 ± 15	<10	<10	<10	<10	<10
	(0/10)	(9/10)	(1/10)	(0/10)	(0/10)	(0/10)	(0/10)	(0/10)
NS5B	<10	80 ± 12 *	60 ± 42	<10	<10	249 ± 61 #	<10	<10
	(0/10)	(10/10)	(4/10)	(0/10)	(0/10)	(10/10)	(0/10)	(0/10)

Four pools of recombinant HCV proteins (described in Materials and Methods) were used as sorbents in ELISA to evaluate antibody production; IgG1 and IgG2a are antibody isotypes. Values show the geometric mean titer ± SD of three measurements done from three independent experiments. * *p* < 0.05 compared to control; **^#^** *p* < 0.05 compared to all groups. Numbers of animals in each group that developed antibodies to the HCV proteins to the total numbers of animals are given in brackets.

**Table 2:** Spontaneous and concanavalin A (ConA)-induced cellular response of lymphocytes from immunized mice in vitro.

Assay	Stimulant	Group 1 mMSC	Group 2 MSC	Group 3 pcNS3-NS5B	Group 4 Control
T-cell proliferation (SI)	ConA	320 ± 22	311 ± 26	348 ± 79	240 ± 17
T-cell proliferation (c.p.m.)	medium	164 ± 46 *	273 ± 84 *	73 ± 15	65 ± 9
	ConA	52406 ± 10014 *	84933 ± 15010 *	25432 ± 12031	15602 ± 7025
ELISA, IFN-γ secretion (pg/mL)	medium	5 ± 2	4 ± 2	4 ± 2	2 ± 2
	ConA	1108 ± 242	1336 ± 380	954 ± 314	902 ± 143
ELISpot, IFN-γ synthesis (SFC/10^6^ cells)	medium	23.0 ± 3.1*	23.5 ± 3.8*	12.4 ± 2.4	10.6 ± 2.2
	ConA	2400 ± 350*	2250 ± 407*	1200 ± 210	750 ± 156

Values are means ± SD of three measurements done in three independent experiments. * *p* < 0.05 compared to Gr3 and Gr4.

## Supplementary Materials

The following are available online at https://www.mdpi.com/2076-393X/8/1/62/s1, Figure S1: Immunohistochemical staining of HCV proteins in MSC transfected with pcNS3-NS5B, Figure S2: Immunoblot analysis of HCV protein expression in MSC transfected with pcNS3-NS5B.
