# Supplementary material for: Genetically Modified Mouse Mesenchymal Stem Cells Expressing Non-Structural Proteins of Hepatitis C Virus Induce Effective Immune Response
Source: Vaccines (Basel). 2020 Feb 2;8(1):62. doi: 10.3390/vaccines8010062 (PMC7158691; doi:10.3390/vaccines8010062)
Supplement: Supplementary file 1 [file vaccines-08-00062-s001.pdf]

Supplementary materials for:

Genetically Modified Mouse Mesenchymal Stem Cells Expressing Non-Structural Proteins of Hepatitis C Virus Induce Effective Immune Response

Olga V. Masalova [1,*], Ekaterina I. Lesnova [1], Regina R. Klimova [1], Ekaterina D. Momotyuk [1], Vyacheslav V. Kozlov [1], Alla M. Ivanova [1], Natalia F. Zakirova [4], Olga V. Payushina [2], Nina N. Butorina [3], Alexander N. Narovlyansky [1], Alexander V. Pronin [1], Alexander V. Ivanov [4,*], and Alla A. Kushch [1]

[1] Gamaleya National Research Center of Epidemiology and Microbiology, Ministry of Health of the Russian Federation, Moscow 123098, Russia; ol.mas@mail.ru (O.V.M.); wolf252006@yandex.ru (E.I.L.); regi.k@mail.ru (R.R.K.); edm95r@rambler.ru (E.D.M.); hyperslava@yandex.ru (V.V.K.); 5893211@bk.ru (A.M.I.); narovl@yandex.ru (A.N.N.); proninalexander@yandex.ru (A.V.P.); vitallku@mail.ru (A.A.K.)

[2] Federal State Autonomous Educational Institution of Higher Education I.M. Sechenov First Moscow State Medical University of the Ministry of Health of the Russian Federation (Sechenov University), Moscow 119991, Russia; payushina@mail.ru (O.V.P.)

[3] Koltzov Institute of Developmental Biology of Russian Academy of Sciences, Moscow 119334, Russia; nnbut@mail.ru (N.N.B.)

[4] Center for Precision Genome Editing and Genetic Technologies for Biomedicine, Engelhardt Institute of Molecular Biology, Russian Academy of Sciences, Moscow 119991, Russia; nat_zakirova@mail.ru (N.F.Z.); aivanov@yandex.ru (A.V.I.)

* Correspondence: ol.mas@mail.ru; Tel.: +7-499-190-30-49 (O.V.M.) or aivanov@yandex.ru; Tel.: +7-199-135-60-65 (A.V.I.)

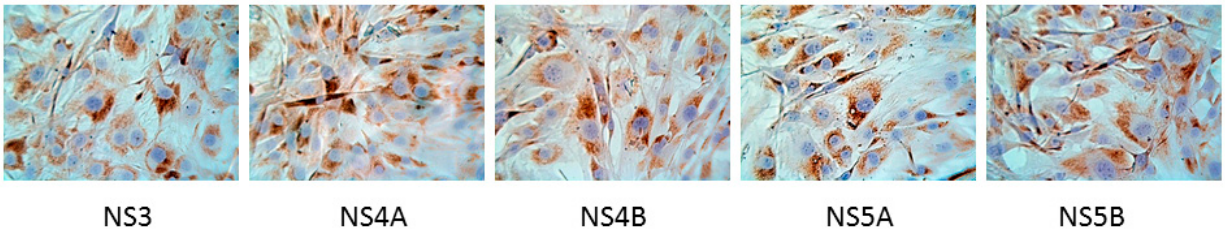

Figure S1. Immunohistochemical staining of HCV proteins in MSC transfected with pcNS3-NS5B 72 h post-transfection. Primary MSC cultures at 3rd-4th passages were transfected with the pcNS3-NS5B plasmid and later selected with G-418. Seventy two hours post-transfection the cells were stained with monoclonal antibodies against HCV proteins using immunoperoxidase method.

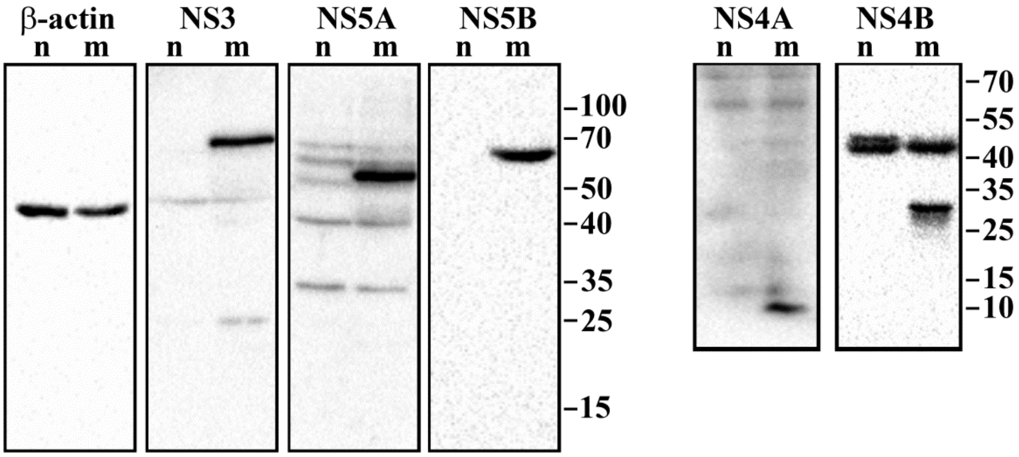

Figure S2. Immunoblot analysis of HCV proteins in MSC transfected with pcNS3-NS5B 72 h post-transfection. The NS3 (70 kDa), NS5A (56 kDa), and NS5B (67 kDa) proteins were resolved on 10% SDS-polyacrylamide gel, whereas the NS4A (6 kDa) and NS4B (27 kDa) proteins - on 15% gel. The proteins were transferred on a nitrocellulose membranes that were inclubated with the respective antibody. In case of NS4B, the membrane was incubated with rabbit anti-NS4B and mice anti-actin antibodies. Letters "n" and "m" denote naive and modified mesenchymal stem cells.
